# Association of nutritional status assessed by the controlling nutritional status score with clinical outcomes in adults with heart failure with preserved ejection fraction

**DOI:** 10.3389/fnut.2026.1940865

**Published:** 2026-09-18

**Authors:** Xin Wang, Zhixin Chen, Fen Wen, Chenyu Guo, Fei Jiang, Jianhua Bi, Danni Wang, Shu Jiang

**Affiliations:** 1 The Third People’s Hospital of Yancheng (The Sixth Affiliated Hospital of Nantong University), Yancheng, China; 2 Jianhu County Center for Disease Control and Prevention, Yancheng, China; 3 The Yancheng Clinical College of Xuzhou Medical University and The First people’s Hospital of Yancheng, Yancheng, China; 4 Department of Epidemiology, School of Public Health, Suzhou Medical College of Soochow University, Suzhou, China; 5 Jiangsu Medical College, Yancheng, China; 6 Department of Health Promotion and Behavioral Sciences, School of Public Health, Anhui Medical University, Hefei, China

**Keywords:** controlling nutritional status score, heart failure with preserved ejection fraction, malnutrition, nutritional assessment, prognosis

## Abstract

**Objective:**

To investigate the association of the controlling nutritional status (CONUT) score with the 1-year risk of readmission and all-cause mortality in patients with heart failure with preserved ejection fraction (HFpEF) after discharge, and to compare its prognostic performance with the geriatric nutritional risk index (GNRI) and the prognostic nutritional index (PNI).

**Methods:**

This multicenter ambispective observational cohort study enrolled adults hospitalized with HFpEF. Patients were stratified according to admission CONUT scores into normal nutrition (0–1 points, *n* = 85), mild risk (2–4 points, *n* = 113), moderate risk (5–8 points, *n* = 79), and severe risk (9–12 points, *n* = 62) groups. The primary endpoints were 1-year readmission and all-cause mortality; cardiogenic shock was evaluated as an exploratory endpoint. Statistical analyses included Kaplan–Meier survival curves, Cox proportional hazards regression models, and receiver operating characteristic (ROC) curves.

**Results:**

A total of 339 patients with HFpEF were analyzed, with a 1-year follow-up completion rate of 96.03%. Higher CONUT risk grades were associated with higher crude rates of 1-year readmission (17.65 to 29.03%, *p* = 0.024) and all-cause mortality (3.53 to 29.03%, *p* < 0.001). Cardiogenic shock occurred in 12 patients (3.54%); its incidence differed across CONUT groups by exact testing (*p* = 0.023), although the number of events was small and the distribution was non-monotonic. In multivariable Cox analysis, the CONUT score was independently associated with 1-year all-cause mortality (HR = 1.48, 95% CI: 1.33–1.65, *p* < 0.001), but not with readmission (HR = 1.05, 95% CI: 0.96–1.16, *p* = 0.281). For all-cause mortality, the AUC of CONUT was 0.760, significantly higher than that of GNRI (AUC = 0.668, *p* < 0.001) but not statistically different from that of PNI (AUC = 0.730, *p* = 0.251). All three tools showed limited discrimination for readmission (AUC range: 0.502–0.526).

**Conclusion:**

The CONUT score was independently associated with 1-year all-cause mortality in adults with HFpEF and demonstrated significantly better discrimination than GNRI. Its discriminative performance was numerically higher than, but not statistically different from, that of PNI. CONUT may therefore serve as a practical supplementary tool for post-discharge risk stratification, although external validation is required.

## 1 Introduction

Heart failure with preserved ejection fraction (HFpEF) represents a major subtype of heart failure, accounting for over 50% of all cases, and its prevalence continues to rise (1–3). The overall prognosis remains suboptimal, characterized by a persistently high risk of mortality (4, 5). Recent evidence highlights nutritional assessment as a critical yet frequently overlooked component in the prognostic management of HFpEF (6). Nutritional impairment is particularly relevant in HFpEF because affected patients are frequently older and multimorbid and may experience chronic systemic inflammation, venous congestion, reduced appetite, impaired gastrointestinal function, and increased catabolic burden. These processes can impair protein synthesis, immune function, and energy metabolism and may further aggravate myocardial remodeling, inflammatory responses, and volume overload (7, 8). Malnutrition was reported in 42% of ambulatory adults with HFpEF despite a high prevalence of obesity (9).

Structured nutritional screening and assessment tools such as Nutrition Risk Screening 2002 and Subjective Global Assessment are used in hospitalized adults (10, 11). Objective nutritional indices, including the prognostic nutritional index (PNI), geriatric nutritional risk index (GNRI), and controlling nutritional status (CONUT) score, are also widely applied in cardiovascular populations (7, 12, 13). These tools capture partially different biological dimensions. PNI integrates serum albumin (Alb) and lymphocyte count (Lym), whereas GNRI combines Alb with an anthropometric component. CONUT incorporates Alb, total cholesterol (TC), and Lym, thereby integrating information related to protein reserve, immune-inflammatory status, and lipid metabolism. In a previous cohort of 371 older adults with HFpEF, 331 (89.2%) had a CONUT score ≥2, illustrating the substantial burden of nutritional risk in this population (13). Because CONUT is calculated from routinely available laboratory parameters, it can generally be derived without additional questionnaires or specialized nutritional assessments (12, 13). However, its components may also be influenced by inflammation, hepatic dysfunction, lipid-lowering therapy, and other non-nutritional factors. Direct comparison of these complementary indices is therefore clinically relevant. Data evaluating the association between CONUT-assessed nutritional status and prognosis in Chinese adults with HFpEF remain limited (14, 15). Accordingly, this multicenter ambispective observational cohort study investigated the association of admission CONUT score with 1-year readmission and all-cause mortality and compared its prognostic performance with PNI and GNRI. Cardiogenic shock was additionally evaluated as a hypothesis-generating exploratory endpoint reflecting severe hemodynamic deterioration; because direct evidence linking nutritional status to cardiogenic shock in HFpEF remains limited, this endpoint was interpreted cautiously.

## 2 Methods

### 2.1 Study population

This multicenter ambispective observational cohort study included patients with index hospitalizations for HFpEF at the First Affiliated Hospital of Soochow University, Yancheng First People’s Hospital, and Peking Union Medical College Hospital between January 2019 and September 2024. The overall observation and outcome-ascertainment period extended through September 2025. Clinical information available before ethics approval was retrospectively extracted from existing medical records, whereas subsequent follow-up and outcome ascertainment were conducted prospectively. The screening workflow is detailed in Figure 1.

**Figure 1:**
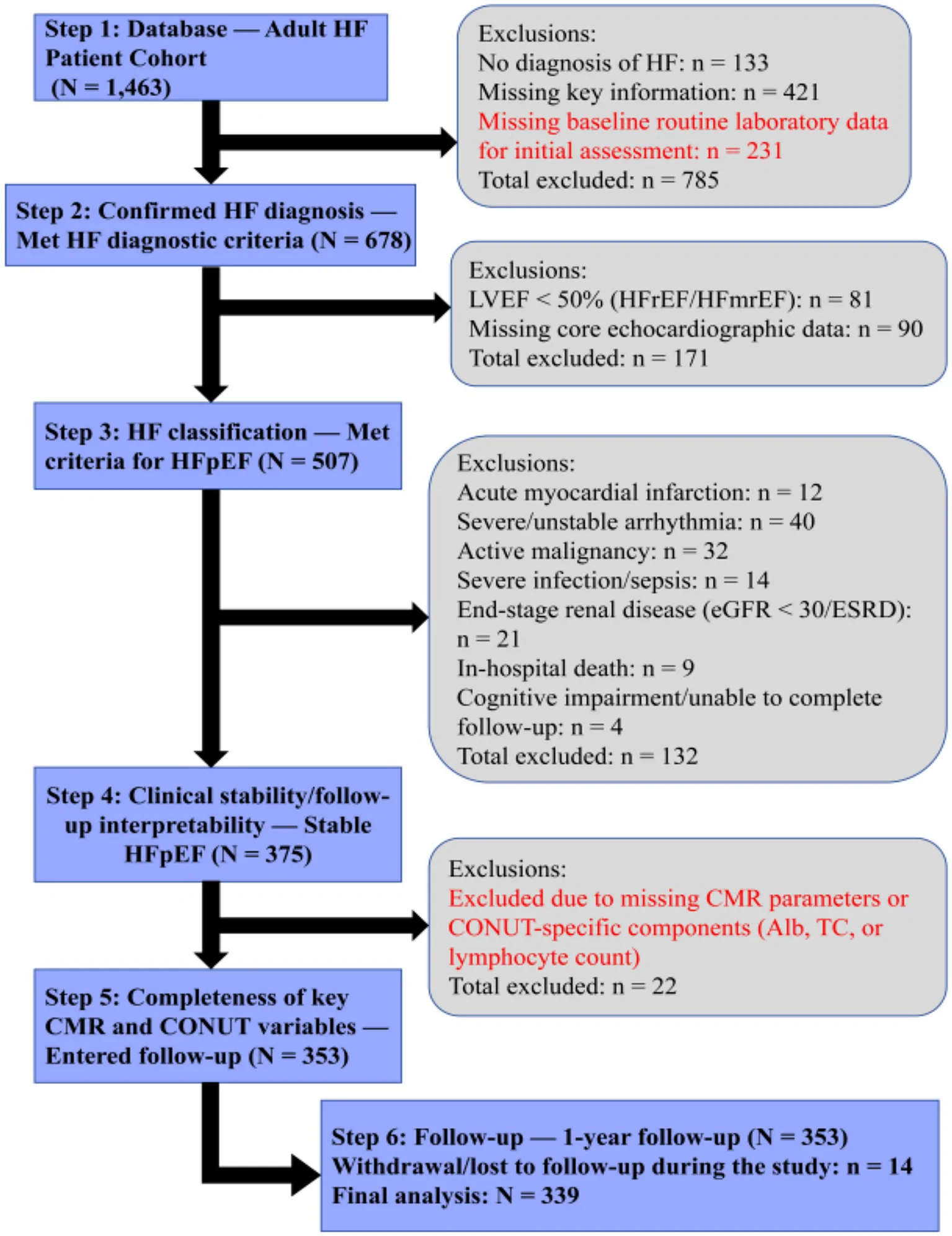
Flowchart of patient screening and enrollment. Exclusions for missing data were applied hierarchically: Step 1 removed patients lacking basic data for initial HF diagnosis and triage; Step 5 further removed otherwise eligible patients with incomplete CONUT score components (albumin, total cholesterol, or lymphocyte count). HF, heart failure; HFrEF, heart failure with reduced ejection fraction; HFmrEF, heart failure with mildly reduced ejection fraction; HFpEF, heart failure with preserved ejection fraction; LVEF, left ventricular ejection fraction; CMR, cardiac magnetic resonance; CONUT, controlling nutritional status; Alb, albumin; TC, total cholesterol; Lym, lymphocyte count; eGFR, estimated glomerular filtration rate; ESRD, end-stage renal disease.

Inclusion criteria were defined as follows: (1) age ≥18 years; (2) meeting the diagnostic criteria for HFpEF according to the 2024 Chinese Guidelines for the Diagnosis and Treatment of Heart Failure (15), defined by the presence of heart failure-related symptoms and/or signs, left ventricular ejection fraction (LVEF) ≥ 50%, and an early mitral inflow velocity to early mitral annular diastolic velocity ratio (E/e′) > 14; (3) N-terminal pro-B-type natriuretic peptide (NT-proBNP) ≥ 400 pg./mL; (4) availability of complete admission laboratory data required for nutritional assessment.

Exclusion criteria comprised: (1) severe coronary artery disease (left main stenosis ≥50% or three-vessel disease with ≥70% stenosis on coronary angiography) or acute myocardial infarction within 12 months prior to admission; (2) moderate or severe valvular stenosis or regurgitation; (3) in-hospital mortality; (4) inability to cooperate or presence of cognitive impairment; (5) contraindications to cardiac magnetic resonance (CMR) imaging (e.g., pacemaker implantation, severe claustrophobia). The study was approved by the Institutional Review Board (IRB) of the Ethics Committee of Yancheng First People’s Hospital on January 12, 2025 (Approval No. 2025-K-033). For the retrospective component, informed consent was waived because only de-identified routine-care data were used; the prospective follow-up phase began only after formal ethical approval.

### 2.2 Data collection and clinical assessment

#### 2.2.1 Baseline characteristics

Demographic characteristics and clinical data, including age, sex, body mass index (BMI), New York Heart Association (NYHA) functional class, and comorbidities, were extracted from the electronic medical record system. Admission laboratory parameters were provided by the department of clinical laboratory. These parameters included Lym, Alb, TC, white blood cell count (WBC), red blood cell count (RBC), hemoglobin (Hb), NT-proBNP, total protein (TP), prealbumin (PA), triglycerides (TG), low-density lipoprotein cholesterol (LDL-C), high-density lipoprotein cholesterol (HDL-C), creatinine (Cr), blood urea nitrogen (BUN), fasting plasma glucose (Glu), potassium, sodium, and phosphorus.

#### 2.2.2 Nutritional assessment

Nutritional status on admission was evaluated using the CONUT, GNRI (16), and PNI (17) tools. The CONUT score was calculated according to the established methodology (18). The specific scoring criteria were as follows: (1) Alb ≥35.0 g/L (0 points), 30.0–34.9 g/L (2 points), 25.0–29.9 g/L (4 points), <25.0 g/L (6 points); (2) Lym ≥ 1.60 × 10^9^/L (0 points), (1.20–1.59) × 10^9^/L (1 point), (0.80–1.19) × 10^9^/L (2 points), <0.80 × 10^9^/L (3 points); (3) TC ≥ 4.65 mmol/L (0 points), 3.62–4.65 mmol/L (1 point), 2.59–3.62 mmol/L (2 points), <2.59 mmol/L (3 points). The sum of these three components yielded the total CONUT score (range: 0–12 points). Based on conventional stratification methods, patients were categorized into a normal nutrition group (0–1 points), a mild nutritional risk group (2–4 points), a moderate nutritional risk group (5–8 points), and a severe nutritional risk group (9–12 points). To maximize the retention of statistical information and avoid data bias resulting from small event sample sizes during multivariable survival analyses, the CONUT, GNRI, and PNI scores were treated as continuous variables during multivariable prognostic model construction. The GNRI was calculated as: GNRI = 1.489 × Alb (g/L) + 41.7 × (BMI/22). The PNI was calculated as: PNI = Alb (g/L) + 5 × Lym (×10^9^/L). BMI was calculated as weight in kilograms divided by the square of height in meters (kg/m^2^).

#### 2.2.3 Follow-up and endpoints

Patients were followed up via outpatient visits or telephone interviews for 1 year after discharge. The final follow-up date was September 30, 2025. The primary endpoints were 1-year readmission and all-cause mortality. Cardiogenic shock was analyzed as an exploratory endpoint. Mortality data were corroborated through medical records or direct confirmation by the patients’ families. Loss to follow-up was defined as the inability to obtain endpoint information within the 1-year observation period.

### 2.3 Statistical analysis

Statistical analyses were performed using R software version 4.3.3. Continuous variables following a normal distribution were presented as mean ± standard deviation and compared using one-way analysis of variance (ANOVA). Non-normally distributed continuous variables were expressed as median (25th percentile, 75th percentile) and analyzed using the Kruskal-Wallis H test. Categorical variables were presented as counts (percentages) and compared using the Chi-square test. Because only 12 cardiogenic shock events occurred and several expected cell counts were small, differences in cardiogenic shock incidence across CONUT groups were evaluated using the Fisher–Freeman–Halton exact test. NT-proBNP exhibited a markedly skewed distribution and underwent Log_10_ transformation prior to analysis.

Kaplan–Meier survival curves were plotted and group differences were assessed using the log-rank test. Univariable Cox proportional hazards models systematically evaluated clinically relevant baseline variables reported in Table 1 for their associations with 1-year readmission and all-cause mortality. Core covariates for multivariable models were not selected solely according to univariable *p* values; selection was guided by established clinical relevance to HFpEF prognosis, prior evidence, availability across participating centers, and the number of endpoint events to limit model overfitting. For the 1-year readmission endpoint, a clinically informed forced-entry adjustment set was chosen to represent complementary prognostic domains: age and BMI for demographic and body-composition characteristics; Log_10_NT-proBNP and NYHA functional class for clinical HF severity; and |GLS| and E/e′ for myocardial deformation and diastolic filling pressure. CONUT was included as the nutritional exposure of interest. Given the 74 readmission events, the model was intentionally kept parsimonious, and variables identified only by univariable statistical significance were not automatically added. Because Hb and serum potassium showed nominal univariable associations with readmission, sensitivity analyses further adjusted the primary readmission model for Hb alone and then for Hb plus serum potassium to assess the robustness of the CONUT estimate. Because only 40 all-cause deaths occurred, a more parsimonious mortality base model comprising age, BMI, Log10NT-proBNP, and creatinine was used. PNI, GNRI, and CONUT were then introduced separately into parallel models to reduce collinearity among nutritional indices. In the primary mortality analyses, CONUT and its individual components (Alb, Lym, and TC) were not entered simultaneously. The component-level sensitivity analysis included Alb, Lym, and TC without CONUT. To assess whether the prognostic performance of CONUT was predominantly attributable to its albumin component, sensitivity analyses compared the base model plus Alb with the base model plus CONUT and with a model containing Alb, Lym, and TC simultaneously. The incremental contribution of Lym and TC beyond Alb was tested using a likelihood-ratio test. Model discrimination and fit were evaluated using the concordance index (C-index) and Akaike information criterion (AIC). Internal validation was conducted using 1,000 bootstrap resamples. Multicollinearity was assessed using the variance inflation factor (VIF), with VIF < 5 indicating no substantial collinearity. Model stability was evaluated by the relative bias of regression coefficients for core predictors, and a penalized C-index was calculated to reflect optimism-corrected performance. The discriminative performance of each nutritional index was evaluated using receiver operating characteristic (ROC) curves, with optimal cutoff values determined by the Youden index. Differences in area under the curve (AUC) were compared using DeLong’s test. All tests were two-sided, with *p* < 0.05 considered statistically significant.

**Table 1:** Baseline characteristics of patients stratified by CONUT score.

Variables	Total (*n* = 339)	Normal nutrition (*n* = 85)	Mild nutritional risk (*n* = 113)	Moderate nutritional risk (*n* = 79)	Severe nutritional risk (*n* = 62)	Test statistic	*p* value
Male sex *n* (%)	182 (53.69)	40 (47.06)	63 (55.75)	47 (59.49)	32 (51.61)	2.191	0.534
Age (years)	55 ± 15	53 ± 14	56 ± 12	55 ± 16	57 ± 15	0.521	0.668
BMI (kg/m^2^)	24.4 ± 4.0	24.9 ± 4.1	24.5 ± 4.4	24.4 ± 3.5	23.9 ± 3.4	0.814	0.487
NYHA functional class *n* (%)							
Class II	222 (65.49)	65 (76.47)	65 (57.52)	55 (69.62)	37 (59.68)	14.210	0.027
Class III	96 (28.32)	18 (21.18)	41 (36.28)	20 (25.32)	17 (27.42)		
Class IV	21 (6.19)	2 (2.35)	7 (6.19)	4 (5.06)	8 (12.90)		
Comorbidities							
Hypertension *n* (%)	137 (40.41)	36 (42.35)	50 (44.25)	31 (39.24)	20 (32.26)	2.580	0.461
Diabetes mellitus *n* (%)	126 (37.17)	33 (38.82)	43 (38.05)	24 (30.38)	26 (41.94)	2.300	0.513
Chronic kidney disease *n* (%)	50 (14.75)	9 (10.59)	11 (9.73)	25 (31.65)	5 (8.06)	8.020	0.046
Chronic obstructive pulmonary disease *n* (%)	79 (23.30)	19 (22.35)	32 (28.32)	13 (16.46)	15 (24.19)	3.733	0.292
|GLS| (%)	14.20 ± 3.01	16.90 ± 3.28	14.10 ± 2.17	13.22 ± 2.19	11.52 ± 2.68	57.106	<0.001
E/e’ ratio	20.08 ± 2.87	20.05 ± 2.91	19.97 ± 2.80	20.04 ± 2.84	20.40 ± 3.03	0.313	0.816
Nutritional parameters							
GNRI	93.08 ± 23.51	114.30 ± 22.06	107.71 ± 21.02	86.68 ± 20.98	75.25 ± 8.80	64.869	<0.001
PNI	43.37 ± 16.00	55.68 ± 13.08	48.81 ± 12.98	34.39 ± 12.82	27.10 ± 5.58	86.999	<0.001
Blood counts							
WBC (×10^9^/L)	7.28 ± 2.81	7.72 ± 2.40	7.64 ± 2.61	7.05 ± 3.35	6.50 ± 2.22	2.950	0.033
Lym (×10^9^/L)	1.66 ± 0.73	2.15 ± 0.68	1.57 ± 0.82	1.50 ± 0.64	1.44 ± 0.63	16.750	<0.001
RBC (×10^12^/L)	4.36 ± 0.76	4.54 ± 0.61	4.30 ± 0.79	4.30 ± 0.77	4.29 ± 0.84	2.530	0.057
Hb (g/L)	130.87 ± 22.49	138.08 ± 18.62	129.71 ± 22.65	128.43 ± 25.45	128.09 ± 21.81	3.680	0.012
TP (g/L)	65.31 (43.12, 86.47)	84.18 (77.29, 91.82)	78.27 (70.26, 83.10)	67.52 (59.74, 74.09)	58.18 (42.33, 64.37)	12.343	<0.001
Log_10_NT-proBNP (pg/mL)	2.93 ± 0.65	2.87 ± 0.62	3.11 ± 0.58	2.93 ± 0.68	2.71 ± 0.73	5.543	<0.001
Biochemical parameters							
Alb (g/L)	35.39 ± 15.67	45.03 ± 13.67	41.41 ± 13.33	27.42 ± 13.56	19.50 ± 3.80	74.027	<0.001
PA (mg/L)	199 (153, 234.50)	223.00 (148.50, 290.50)	212.00 (125.50, 255.00)	198 (99, 251.00)	130 (80, 187)	141.440	<0.001
TG (mmol/L)	1.54 (1.13, 2.45)	2.90 (2.25, 3.15)	1.81 (1.34, 2.53)	1.43 (1.14, 1.99)	1.20 (0.97, 1.65)	77.449	<0.001
TC (mmol/L)	3.19 (1.73, 4.26)	3.92 (1.46, 4.62)	3.23 (2.83, 4.80)	3.22 (2.76, 3.51)	1.25 (1.09, 1.49)	53.450	<0.001
HDL-C (mmol/L)	0.93 (0.85, 1.07)	0.98 (0.89, 1.18)	0.95 (0.80, 1.12)	0.89 (0.85, 1.01)	0.89 (0.69, 0.97)	24.856	<0.001
LDL-C (mmol/L)	2.31 (1.72, 3.45)	2.31 (2.31, 3.45)	2.31 (1.64, 2.58)	2.31 (1.76, 3.45)	2.05 (1.42, 3.34)	16.756	<0.001
Glu (mmol/L)	8.40 (4.82, 9.75)	5.60 (4.65, 6.60)	8.25 (5.50, 9.90)	9.06 (6.37, 10.10)	9.74 (6.20, 10.80)	31.274	<0.001
BUN (mmol/L)	4.49 (3.91, 6.42)	4.79 (4.30, 6.62)	6.00 (4.58, 8.42)	4.11 (3.61, 4.62)	3.95 (3.72, 4.22)	26.514	<0.001
Cr (μmol/L)	71.00 (59.00, 85.00)	68.80 (57.00, 78.00)	71.00 (59.00, 86.00)	71.00 (59.85, 86.35)	71.00 (68.00, 85.00)	4.675	0.197
Potassium (mmol/L)	3.93 ± 0.56	3.97 ± 0.41	3.93 ± 0.55	3.88 ± 0.74	3.77 ± 0.43	1.522	0.209
Phosphorus (mmol/L)	1.28 ± 0.20	1.28 ± 0.20	1.28 ± 0.20	1.27 ± 0.18	1.29 ± 0.24	0.055	0.983
Sodium (mmol/L)	139.21 ± 2.83	139.41 ± 2.93	138.91 ± 2.73	139.12 ± 2.88	139.73 ± 2.79	0.702	0.552
Outcomes							
Readmission *n* (%)	74 (21.83)	15 (17.65)	21 (18.58)	20 (25.32)	18 (29.03)	9.474	0.024
All-cause mortality *n* (%)	40 (11.80)	3 (3.53)	11 (9.73)	8 (10.13)	18 (29.03)	28.372	<0.001
Cardiogenic shock *n* (%)	12 (3.54)	1 (1.18)	4 (3.54)	7 (8.86)	0 (0.00)	—	0.023

Alb albumin, BMI body mass index, BUN blood urea nitrogen, CONUT controlling nutritional status, Cr creatinine, E/e’ ratio of early diastolic transmitral flow velocity to early diastolic mitral annular velocity, GLS global longitudinal strain, Glu fasting plasma glucose, GNRI geriatric nutritional risk index, Hb hemoglobin, HDL-C high-density lipoprotein cholesterol, IQR interquartile range, LDL-C low-density lipoprotein cholesterol, Lym lymphocyte, NT-proBNP N-terminal pro-B-type natriuretic peptide, NYHA New York Heart Association, PA prealbumin, PNI prognostic nutritional index, RBC red blood cell count, SD standard deviation, TC total cholesterol, TG triglycerides, TP total protein, WBC white blood cell count. For cardiogenic shock, the P value was obtained using the Fisher–Freeman–Halton exact test because of the small number of events.

## 3 Results

### 3.1 Baseline characteristics

The 1-year follow-up completion rate was 96.03% (339/353). Baseline characteristics did not differ significantly between patients lost to follow-up and the 339 analyzed patients (*p* > 0.05). Based on CONUT scores, patients were categorized into normal nutrition (*n* = 85), mild risk (*n* = 113), moderate risk (*n* = 79), and severe risk (*n* = 62) groups. Higher CONUT scores correlated with progressive declines in core nutritional and metabolic parameters, including GNRI (*F* = 64.869, *p* < 0.001), PNI (*F* = 86.999, *p* < 0.001), Alb (*F* = 74.027, *p* < 0.001), TC (H = 53.450, *p* < 0.001), and Lym (*F* = 16.750, *p* < 0.001). Regarding cardiac function and clinical features, significant intergroup differences were observed for chronic kidney disease prevalence (*χ*^2^ = 8.020, *p* = 0.046), NYHA functional class (*χ*^2^ = 14.210, *p* = 0.027), Hb (*F* = 3.680, *p* = 0.012), Log_10_ NT-proBNP (*F* = 5.543, *p* < 0.001), and the absolute value of global longitudinal strain (|GLS|) (*F* = 57.106, *p* < 0.001). No significant differences were noted in the E/e’ ratio or the prevalence of hypertension and diabetes mellitus across the groups (all *p* > 0.05) (Table 1).

### 3.2 Clinical outcomes during follow-up

During the 1-year follow-up, the 339 patients with HFpEF experienced 74 readmissions (21.83%), 40 all-cause deaths (11.80%), and 12 episodes of cardiogenic shock (3.54%) (Table 1). Readmission rates increased from 17.65% in the normal nutrition group and 18.58% in the mild-risk group to 29.03% in the severe-risk group (*p* = 0.024). All-cause mortality was 3.53% in the normal nutrition group and 29.03% in the severe-risk group (*p* < 0.001). Cardiogenic shock occurred in 1.18, 3.54, 8.86, and 0% of the normal, mild, moderate, and severe nutritional-risk groups, respectively. Given the small number of events, exact testing was used and demonstrated an overall intergroup difference (*p* = 0.023); however, the non-monotonic distribution warrants cautious interpretation. Kaplan–Meier survival analysis and log-rank testing showed significant differences in the cumulative incidence of 1-year readmission, all-cause mortality, and cardiogenic shock across CONUT groups (*χ*^2^ values: 15.769, 42.387, and 14.211, respectively; all *p* < 0.01) (Figure 2).

**Figure 2:**
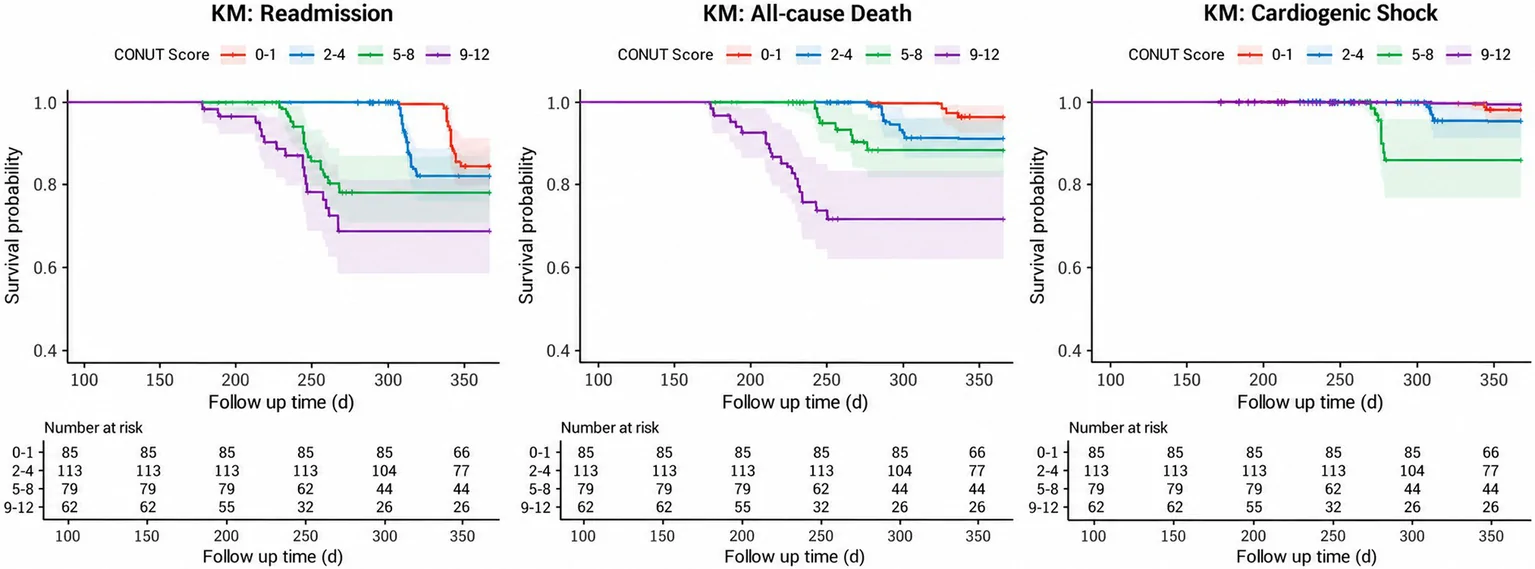
Kaplan–Meier survival curves stratified by CONUT score groups.

### 3.3 Cox proportional hazards regression analysis

#### 3.3.1 One-year readmission risk

Univariable Cox regression identified lower BMI (HR = 0.92, 95% CI: 0.87–0.98, *p* = 0.010), NYHA functional class III (HR = 1.76, 95% CI: 1.08–2.86, *p* = 0.023), higher Log10NT-proBNP (HR = 1.67, 95% CI: 1.15–2.43, *p* = 0.007), lower |GLS| (HR = 0.89, 95% CI: 0.82–0.96, *p* = 0.002), lower Hb (HR = 0.99, 95% CI: 0.98–1.00, *p* = 0.031), and lower serum potassium (HR = 0.60, 95% CI: 0.36–1.00, *p* = 0.048) as factors associated with 1-year readmission; the complete univariable results, including HDL-C, LDL-C, Glu, and BUN, are shown in Table 2. In the primary multivariable model, the CONUT score was not independently associated with 1-year readmission (HR = 1.05, 95% CI: 0.96–1.16, *p* = 0.281). Lower BMI remained independently associated with higher readmission risk (HR = 0.93, 95% CI: 0.87–1.00, *p* = 0.040), whereas the association with Log_10_NT-proBNP was attenuated after adjustment (HR = 1.50, 95% CI: 0.99–2.28, *p* = 0.056). Multicollinearity diagnostics (VIF < 4.0) and bootstrap internal validation supported the stability of the model, although some optimism remained (apparent C-index: 0.668; penalized C-index: 0.628) (Table 2). In sensitivity analyses, additional adjustment for Hb alone did not materially alter the association between CONUT and readmission (HR = 1.05, 95% CI: 0.96–1.16, *p* = 0.298); the result was similarly unchanged after further adjustment for serum potassium (HR = 1.05, 95% CI: 0.96–1.16, *p* = 0.295).

**Table 2:** Cox proportional hazards regression analysis of factors associated with 1-year readmission in patients with HFpEF.

Variables	Univariable *HR* (95%*CI*)	*p* value	Multivariable *HR* (95% *CI*)	*p* value
Male sex (vs. female)	0.72 (0.46–1.14)	0.157	—	—
Age (years)	1.02 (1.00–1.03)	0.061	1.01 (0.99–1.03)	0.309
BMI (kg/m^2^)	0.92 (0.87–0.98)	0.010	0.93 (0.87–1.00)	0.040
PNI	0.99 (0.97–1.01)	0.262	—	—
GNRI	0.99 (0.98–1.00)	0.138	—	—
CONUT score	1.06 (0.98–1.14)	0.127	1.05 (0.96–1.16)	0.281
Log_10_NT-proBNP (pg/mL)	1.67 (1.15–2.43)	0.007	1.50 (0.99–2.28)	0.056
NYHA functional class (vs. Class II)				
Class III	1.76 (1.08–2.86)	0.023	0.98 (0.45–2.13)	0.957
Class IV	2.21 (0.94–5.20)	0.071	0.75 (0.19–2.95)	0.685
|GLS| (%)	0.89 (0.82–0.96)	0.002	0.90 (0.78–1.04)	0.170
E/e’ ratio	0.97 (0.90–1.05)	0.445	0.97 (0.89–1.05)	0.382
Hypertension	0.80 (0.50–1.28)	0.351	—	—
Diabetes mellitus	1.19 (0.75–1.89)	0.463	—	—
Chronic kidney disease	0.90 (0.46–1.75)	0.755	—	—
Chronic obstructive pulmonary disease	0.72 (0.40–1.29)	0.275	—	—
WBC (×10^9^/L)	1.00 (0.93–1.09)	0.926	—	—
Lym (×10^9^/L)	0.71 (0.50–1.00)	0.053	—	—
RBC (×10^12^/L)	0.84 (0.63–1.11)	0.222	—	—
Hb (g/L)	0.99 (0.98–1.00)	0.031	—	—
TP (g/L)	1.00 (0.99–1.01)	0.870	—	—
Alb (g/L)	0.99 (0.98–1.01)	0.491	—	—
PA (mg/L)	0.99 (0.99–1.00)	0.406	—	—
Potassium (mmol/L)	0.60 (0.36–1.00)	0.048	—	—
Phosphorus (mmol/L)	1.02 (0.34–3.09)	0.972	—	—
Sodium (mmol/L)	1.01 (0.93–1.10)	0.818	—	—
TG (mmol/L)	0.98 (0.78–1.23)	0.833	—	—
TC (mmol/L)	1.10 (0.94–1.28)	0.229	—	—
HDL-C (mmol/L)	0.92 (0.37–2.30)	0.856	—	—
LDL-C (mmol/L)	0.93 (0.81–1.06)	0.283	—	—
Glu (mmol/L)	1.001 (0.997–1.005)	0.636	—	—
BUN (mmol/L)	0.99 (0.92–1.07)	0.856	—	—
Cr (μmol/L)	1.00 (0.99–1.01)	0.590	—	—

“—” indicates that the variable was not included in the multivariable analysis model.

#### 3.3.2 One-year all-cause mortality risk

In separate univariable Cox models, older age, elevated Log_10_NT-proBNP, NYHA functional class IV, reduced |GLS|, lower PNI and GNRI, higher CONUT score, lower Lym, RBC, Hb, TP, Alb, PA, TC, and HDL-C, and higher Glu were associated with 1-year all-cause mortality (all *p* < 0.05; Table 3). These estimates represent separate unadjusted associations and should not be interpreted as mutually independent effects, particularly for CONUT and its constituent variables. Given the limited number of mortality events and consequent constraints on model degrees of freedom, a parsimonious base model (Model 1) comprising age, BMI, Log_10_NT-proBNP, and Cr was constructed. PNI, GNRI, and CONUT were then introduced separately into parallel models. In the CONUT model, the CONUT score remained independently associated with 1-year all-cause mortality (HR = 1.48, 95% CI: 1.33–1.65, *p* < 0.001). The CONUT model showed the highest discrimination and best fit among the parallel nutritional models (C-index 0.853; AIC 387.19), compared with the PNI model (C-index 0.814; AIC 407.44) and the GNRI model (C-index 0.797; AIC 413.82). Collinearity diagnostics (all VIF < 4.0) and bootstrap internal validation supported the stability of the parsimonious models, while the limited number of deaths was considered when interpreting model performance (Table 3).

**Table 3:** Cox proportional hazards regression analysis of factors associated with 1-year all-cause mortality in patients with HFpEF.

Variables	Univariable *HR* (95% *CI*)	*p* value	Model 1 *HR* (95% *CI*)	*p* value	Model 1 + PNI *HR* (95% *CI*)	*p* value	Model 1 + GNRI *HR* (95% CI)	*p* value	Model 1 + CONUT *HR* (95% *CI*)	*p* value
Male sex (vs. female)	1.16 (0.62–2.17)	0.646	—	—	—	—	—	—	—	—
Age (years)	1.03 (1.01–1.05)	0.012	1.03 (1.01–1.05)	0.028	1.02 (1.00–1.05)	0.077	1.03 (1.00–1.05)	0.045	1.03 (1.01–1.06)	0.017
BMI (kg/m^2^)	1.03 (0.95–1.11)	0.490	1.07 (0.99–1.16)	0.093	1.09 (1.00–1.18)	0.048	1.20 (1.09–1.31)	<0.001	1.09 (0.99–1.19)	0.068
PNI	0.93 (0.91–0.96)	<0.001	—	—	0.92 (0.89–0.95)	<0.001	—	—	—	—
GNRI	0.97 (0.95–0.98)	<0.001	—	—	—	—	0.95 (0.93–0.97)	<0.001	—	—
CONUT score	1.38 (1.24–1.52)	<0.001	—	—	—	—	—	—	1.48 (1.33–1.65)	<0.001
Log_10_NT-proBNP (pg/mL)	2.57 (1.52–4.34)	<0.001	2.53 (1.47–4.34)	<0.001	2.90 (1.73–4.87)	<0.001	3.16 (1.88–5.33)	<0.001	3.45 (2.06–5.78)	<0.001
NYHA functional class (vs. Class II)										
Class III	1.88 (0.96–3.67)	0.065	—	—	—	—	—	—	—	—
Class IV	3.51 (1.31–9.36)	0.012	—	—	—	—	—	—	—	—
|GLS| (%)	0.81 (0.74–0.90)	<0.001	—	—	—	—	—	—	—	—
E/e’ ratio	1.02 (0.91–1.13)	0.776	—	—	—	—	—	—	—	—
Hypertension	0.61 (0.31–1.20)	0.150	—	—	—	—	—	—	—	—
Diabetes mellitus	0.71 (0.36–1.39)	0.318	—	—	—	—	—	—	—	—
Chronic kidney disease	1.63 (0.78–3.42)	0.198	—	—	—	—	—	—	—	—
Chronic obstructive pulmonary disease	0.92 (0.44–1.94)	0.832	—	—	—	—	—	—	—	—
WBC (×10^9^/L)	0.88 (0.77–1.01)	0.068	—	—	—	—	—	—	—	—
Lym (×10^9^/L)	0.38 (0.22–0.63)	<0.001	—	—	—	—	—	—	—	—
RBC (×10^12^/L)	0.65 (0.45–0.94)	0.021	—	—	—	—	—	—	—	—
Hb (g/L)	0.99 (0.97–1.00)	0.044	—	—	—	—	—	—	—	—
TP (g/L)	0.97 (0.96–0.99)	<0.001	—	—	—	—	—	—	—	—
Alb (g/L)	0.94 (0.91–0.97)	<0.001	—	—	—	—	—	—	—	—
PA (mg/L)	0.995 (0.992–0.998)	0.003	—	—	—	—	—	—	—	—
Potassium (mmol/L)	0.98 (0.57–1.71)	0.952	—	—	—	—	—	—	—	—
Phosphorus (mmol/L)	0.43 (0.09–2.13)	0.299	—	—	—	—	—	—	—	—
Sodium (mmol/L)	1.10 (0.98–1.25)	0.104	—	—	—	—	—	—	—	—
TG (mmol/L)	1.22 (0.95–1.56)	0.120	—	—	—	—	—	—	—	—
TC (mmol/L)	0.75 (0.59–0.94)	0.014	—	—	—	—	—	—	—	—
HDL-C (mmol/L)	0.21 (0.05–0.83)	0.025	—	—	—	—	—	—	—	—
LDL-C (mmol/L)	0.94 (0.78–1.13)	0.514	—	—	—	—	—	—	—	—
Glu (mmol/L)	1.004 (1.001–1.007)	0.006	—	—	—	—	—	—	—	—
BUN (mmol/L)	0.91 (0.79–1.05)	0.197	—	—	—	—	—	—	—	—
Cr (μmol/L)	1.00 (1.00–1.01)	0.254	1.00 (0.99–1.01)	0.858	1.00 (0.99–1.01)	0.650	1.00 (0.99–1.01)	0.775	1.00 (0.99–1.01)	0.807
C-index	—	—	0.680	—	0.814	—	0.797	—	0.853	—
AIC	—		441.32		407.44		413.82		387.19	

Each univariable HR was estimated from a separate Cox model and represents an unadjusted association. Because CONUT and its components (Alb, Lym, and TC), as well as the other nutritional indices, are biologically and mathematically related, the univariable estimates should not be interpreted as mutually independent effects. PNI, GNRI, and CONUT were entered separately into the primary multivariable models. CONUT and Alb/Lym/TC were not entered simultaneously in the primary CONUT model; the component-level sensitivity model included Alb, Lym, and TC without CONUT.

#### 3.3.3 Sensitivity analysis of the contribution of serum albumin to CONUT

To determine whether the prognostic performance of CONUT was predominantly attributable to its Alb component, additional sensitivity analyses were performed using the same mortality base model. Adding Alb alone yielded a C-index of 0.797 and an AIC of 413.82; Alb remained independently associated with mortality (HR = 0.92, 95% CI: 0.89–0.95, *p* < 0.001). In comparison, the CONUT model achieved a C-index of 0.853 and an AIC of 387.19 (HR = 1.48, 95% CI: 1.33–1.65, *p* < 0.001). When Alb, Lym, and TC were entered simultaneously, all three components remained independently associated with mortality, and the model achieved a C-index of 0.840 and an AIC of 400.12. Addition of Lym and TC significantly improved model fit beyond Alb alone (likelihood-ratio χ^2^ = 17.68, df = 2, *p* < 0.001), indicating that the prognostic information captured by CONUT was not attributable solely to Alb (Table 4).

**Table 4:** Sensitivity analysis assessing whether the prognostic value of CONUT is predominantly attributable to serum albumin.

Model	Additional predictor(s)	Adjusted HR (95% CI)	*p* value	C-index	AIC
Model 1	—	—	—	0.680	441.32
Model 1 + Alb	Alb	0.92 (0.89–0.95)	<0.001	0.797	413.82
Model 1 + CONUT	CONUT	1.48 (1.33–1.65)	<0.001	0.853	387.19
Model 1 + Alb + Lym + TC	Alb	0.93 (0.90–0.96)	<0.001	0.840	400.12
	Lym	0.36 (0.20–0.64)	<0.001	—	—
	TC	0.69 (0.54–0.89)	0.004	—	—

Model 1 included age, BMI, Log_10_NT-proBNP, and Cr. Compared with Model 1 + Alb, addition of Lym and TC significantly improved model fit (likelihood-ratio *χ*^2^ = 17.68, df = 2, *p* < 0.001). Alb, albumin; Lym, lymphocyte count; TC, total cholesterol.

### 3.4 Prognostic performance of three nutritional tools

ROC analysis revealed that the CONUT score possessed favorable predictive value for 1-year all-cause mortality in HFpEF patients, presenting an AUC of 0.760 (95%CI 0.677–0.842, *p* < 0.001) with an optimal cutoff of 6.50. The PNI had an AUC of 0.730 (95%CI 0.638–0.822, *p* < 0.001), and the GNRI achieved an AUC of 0.668 (95% CI 0.565–0.770, *p* < 0.001). The DeLong test verified that the predictive value of the CONUT score for all-cause mortality was significantly superior to the GNRI (*p* < 0.001). While the AUC of CONUT was numerically higher than that of PNI, the difference was not statistically significant (*p* = 0.251). Conversely, all three tools demonstrated limited capacity to predict 1-year readmission. Their respective AUCs were 0.502 (CONUT), 0.526 (PNI), and 0.503 (GNRI), with none reaching statistical significance (all *p* > 0.05). The DeLong test indicated no significant differences in readmission predictive performance among the three indices (all *p* > 0.05) (Figure 3; Table 5).

**Figure 3:**
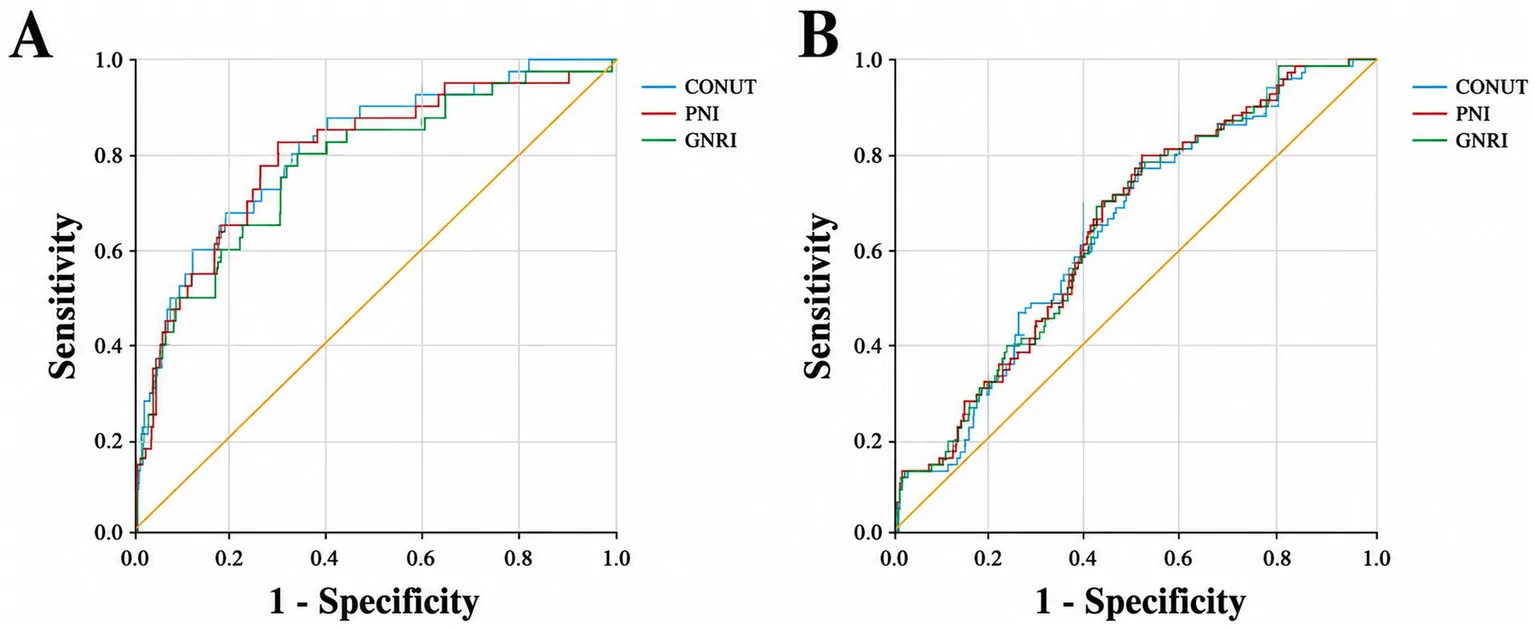
ROC curves comparing the prognostic capability of CONUT, PNI, and GNRI for adverse outcomes in HFpEF patients (translation omitted). **(A)** All-cause mortality prediction; **(B)** Readmission prediction.

**Table 5:** Predictive performance of CONUT, PNI, and GNRI for adverse outcomes.

Nutritional assessment tool	AUC	95% *CI*	*p* value	Sensitivity	Specificity	*p* value (vs. CONUT)	*p* value (vs. GNRI)	*p* value (vs. PNI)	Cut-off value
All-cause mortality									
CONUT	0.760	0.677–0.842	<0.001	62.5%	78.9%	Ref	<0.001	0.251	>6.50
GNRI	0.668	0.565–0.770	<0.001	57.5%	77.3%	<0.001	Ref	0.383	<83.39
PNI	0.730	0.638–0.822	<0.001	60.0%	79.9%	0.251	0.383	Ref	<32.67
Readmission									
CONUT	0.502	0.431–0.572	0.952	83.8%	21.9%	Ref	0.364	0.946	>0.50
GNRI	0.526	0.452–0.599	0.475	83.8%	26.8%	0.364	Ref	0.411	<110.01
PNI	0.503	0.428–0.578	0.934	45.9%	61.1%	0.946	0.411	Ref	<39.80

## 4 Discussion

Previous studies have established the prognostic relevance of malnutrition in HFpEF (14, 15), yet direct comparisons of nutritional tools across specific post-discharge outcomes remain limited. In this multicenter ambispective cohort, higher CONUT risk grades were associated with higher crude rates of readmission and all-cause mortality. After multivariable adjustment, however, CONUT remained independently associated with 1-year all-cause mortality but not with readmission. ROC analysis further showed that CONUT discriminated all-cause mortality significantly better than GNRI, whereas its AUC was numerically higher than, but not statistically different from, that of PNI. The non-significant DeLong comparison with PNI should not be interpreted as evidence of equivalence, particularly given the modest number of mortality events.

The observed difference between CONUT and GNRI may partly reflect the biological domains represented by the two indices. Both incorporate Alb, but GNRI additionally incorporates an anthropometric component, whereas CONUT incorporates Lym and TC. CONUT may therefore capture immune-inflammatory and lipid-metabolic abnormalities in addition to protein depletion, while anthropometric measures in HFpEF may be influenced by obesity and fluid status. The additional sensitivity analysis also indicated that the prognostic information in CONUT was not attributable to Alb alone: although Alb was strongly associated with mortality, adding Lym and TC significantly improved model fit beyond the Alb-only model (likelihood-ratio *χ*^2^ = 17.68, df = 2, *p* < 0.001). These findings support the complementary information provided by the three CONUT components but should not be interpreted as evidence that CONUT is universally superior to other nutritional indices.

Baseline analysis revealed that NT-proBNP levels in the severe nutritional-risk group (CONUT score 9–12) were paradoxically lower than those in some of the less severe nutritional risk groups. This non-linear pattern is noteworthy but should be interpreted with caution, as the present study was not designed to determine its underlying mechanism. Several factors may contribute to this observed pattern. NT-proBNP concentrations in HFpEF can vary according to disease phenotype, body composition, volume status, renal function, atrial rhythm, and overall disease severity (19–24). Atrial fibrillation can independently influence NT-proBNP concentrations in patients with heart failure (25). In addition, exclusion of patients who died during the index hospitalization may have introduced selection effects (26), potentially under-representing the most critically ill patients with different NT-proBNP profiles. Because detailed measures of volume status and natriuretic peptide physiology were not available, and atrial fibrillation was not collected as a dedicated variable across participating centers, these potential explanations cannot be distinguished in the present cohort. Accordingly, the observed NT-proBNP pattern should be regarded as hypothesis-generating rather than evidence of a specific biological mechanism. HFpEF is characterized by systemic inflammatory and metabolic heterogeneity (27–29), while the three CONUT components reflect complementary protein/inflammatory, immune, and lipid-metabolic domains (30–32). Additional microvascular and inflammatory mechanisms are also central to HFpEF pathobiology (33, 34). Future studies integrating serial nutritional assessments, comprehensive biomarker profiling, and detailed comorbidity characterization are needed to clarify the interplay between nutritional status and natriuretic peptide dynamics in this population.

A prognostic divergence was observed in the present study: all three nutritional tools showed useful discrimination for 1-year all-cause mortality but limited ability to discriminate readmission. This pattern suggests that mortality may be more closely related to systemic nutritional, inflammatory, and metabolic vulnerability, whereas readmission is also strongly influenced by factors such as acute volume overload, comorbidities, treatment, and medication adherence (35). Cause of death was not systematically adjudicated across participating centers; therefore, the present study cannot determine whether the association between CONUT and mortality predominantly reflects cardiovascular vulnerability or broader systemic processes such as infection, inflammation, cachexia, or frailty. Accordingly, the prognostic association of CONUT in this cohort should be interpreted as reflecting a composite of cardiovascular and systemic vulnerability rather than a purely cardiac-specific signal. Similarly, because cardiogenic shock events were few and showed a non-monotonic distribution across CONUT categories, the exploratory shock findings should not be used to infer the mechanism of death. In clinical practice, CONUT is based on routinely measured laboratory parameters and may therefore provide a practical supplementary method for risk stratification. Its use may help identify patients who warrant more comprehensive nutritional and clinical assessment, although whether CONUT-guided intervention improves outcomes requires prospective evaluation (35–37).

Several limitations should be acknowledged. First, the observational design precludes causal inference, and residual confounding cannot be excluded. Second, follow-up was limited to 1 year and nutritional status was assessed primarily at baseline; therefore, the prognostic implications of longitudinal changes in nutritional status could not be evaluated. Third, important treatment-related and socioeconomic factors, including contemporary HFpEF pharmacotherapy such as SGLT2 inhibitors, were not comprehensively available and could not be fully adjusted for. Fourth, the numbers of all-cause deaths and other adverse events were relatively limited. Although parsimonious models and bootstrap internal validation were used to reduce model optimism, overfitting cannot be completely excluded. Fifth, the prognostic models were internally validated only; their discrimination, calibration, and proposed cutoff values require confirmation in independent external cohorts. Sixth, because patients who died during the index hospitalization were excluded to specifically evaluate post-discharge outcomes, survivor bias may have occurred, potentially under-representing the most critically ill patients. Seventh, cause-specific mortality was not systematically adjudicated, precluding separate evaluation of cardiovascular and non-cardiovascular death and limiting our ability to determine whether the association between CONUT and mortality was predominantly cardiovascular or reflected broader systemic vulnerability. Finally, atrial fibrillation was not collected as a dedicated variable across participating centers, precluding reliable reconstruction of its prevalence or adjustment for this potential confounder. Because atrial fibrillation can independently influence NT-proBNP concentrations, residual confounding of analyses involving NT-proBNP cannot be excluded.

## 5 Conclusion

In conclusion, the CONUT score was independently associated with 1-year all-cause mortality in adults with HFpEF. It demonstrated significantly better discrimination than GNRI, whereas its discriminative performance was numerically higher but not statistically different from that of PNI. Sensitivity analysis suggested that the prognostic information provided by CONUT was not explained by serum albumin alone. CONUT may therefore serve as a practical supplementary tool for clinical risk stratification in HFpEF, although independent external validation is required before broader application.

## Data Availability

The datasets presented in this article are not readily available because the dataset contains sensitive clinical information of enrolled patients. It is restricted by the institutional ethics committee and local personal privacy regulations to protect patient confidentiality, thus cannot be publicly shared in full. De-identified partial data can be provided by the corresponding author upon reasonable academic request. Requests to access the datasets should be directed to SJ, 63461800@qq.com.
